# Psychometric properties of the Chinese version of the spiritual care-giving scale (C-SCGS) in nursing practice

**DOI:** 10.1186/s12874-019-0662-7

**Published:** 2019-01-23

**Authors:** Yanli Hu, Lay Hwa Tiew, Fan Li

**Affiliations:** 1 School of Nursing, Jilin University, Changchun, China; 2 National University Hospital, Alice Lee Centre for Nursing Studies, National University of Singapore, Singapore, Singapore; 3 Department of Pathogenobiology, The Key Laboratory of Zoonosis research, Chinese Ministry of Education, College of Basic Medicine, Jilin University, No.126 Xinmin Street, Changchun, China; 4 The Key Laboratory for Bionics Engineering, Ministry of Education, Jilin University, Changchun, China; 5 State Key Laboratory of Pathogenesis, Prevention and Treatment of High Incidence Diseases in Central Asia, Urumqi, China

**Keywords:** Psychometrics, Spiritual care, Spirituality, Nurses

## Abstract

**Background:**

Spiritual care is defined as recognizing and responding to the needs of the human spirit when the individual is facing trauma, illness, or sadness. Providing spiritual care is one of the core aspects of holistic care, as it is significantly associated with patients’ quality of life. The provision of optimal spiritual care requires good understanding by the nurses. Therefore, it is important to assess this understanding by using a proven, well-validated instrument. The Spiritual Care-Giving Scale (SCGS) is designed to measure nurses’ perceptions of spirituality and spiritual care in Singapore. However, it is unknown whether this scale is valid and reliable for use with nurses in the context of Chinese culture. The purpose of this study is to determine this version’s validity and reliability for use with nurses in China.

**Methods:**

In this quantitative, descriptive, cross-sectional study, after translating the English version of the SCGS into Chinese and making adjustments for culture and values, we assessed the performance of this instrument by administering the C-SCGS to a convenience sample of 400 nurses. The participants also completed the Chinese version of the Spiritual Care Competency Scale (C-SCCS) to assess the concurrent validity of the C-SCGS. The internal consistency and homogeneity of the C-SCGS were also tested, and a factorial analysis was performed.

**Results:**

Completed questionnaires were obtained from 355 participants (response rate: 88.75%). Four factors were confirmed by an exploratory factor analysis (EFA) using Promax with the Kaiser normalization rotation method after the 35-item SCGS was reduced to 34 items, and these factors explained 53.116% of the total variance. The adjusted item–total correlation ranged from 0.527 to 0.760. The Cronbach’s alpha of the factors ranged from 0.836 to 0.941, and the Guttman split-half coefficient was between 0.759 and 0.902. The concurrent validity of the C-SCGS and C-SCCS (r = 0.534, *p* < 0.01) showed a significant correlation. Nurses’ education showed a significant association with the scores of the C-SCGS.

**Conclusion:**

The C-SCGS was found to be a psychometrically sound measurement to evaluate Chinese-speaking nurses’ perceptions of spirituality and spiritual care.

**Electronic supplementary material:**

The online version of this article (10.1186/s12874-019-0662-7) contains supplementary material, which is available to authorized users.

## Background

Spiritual well-being is an important component and predictor of patient health-related quality of life for individuals with chronic or severe diseases. Spiritual care is defined as recognizing and responding to the needs of the human spirit when the individual is facing trauma, illness, or sadness. It can include addressing the need for satisfactory meaning, self-worth, self-expression, the support of faith, the practice of rituals, prayers or sacraments, and conversation with sensitive listeners. Spiritual care is considered an essential part of palliative care and holistic care [1–7] because it can help enhance the physical, social, and psychological aspects of good health in patients by reducing tension and stress, increasing support and adaptation abilities, maintaining hope, and helping patients find meaning and purpose [1, 8–11]. Optimal spiritual care requires good understanding on the part of nurses [12–15] to allow them to discern what action is required with regard to the spiritual aspect of nursing practice and enable them to explore the resources available to improve patient care and enhance patient satisfaction [15–19]. A number of instruments have been developed to evaluate nurses’ views on spirituality and spiritual care in several countries, such as the Spirituality and Spiritual Care Rating Scale (SSCRS) [20] and the Spiritual Care-Giving Scale (SCGS) [21]. However, such instruments are lacking in China. Although the C-SCCS was used for evaluation in this study, it is designed to measure the spiritual care capacity of clinical caregivers, and there is currently a lack of effective tools for assessing the spirituality and spiritual care perceptions of nurses. This issue needs to be addressed urgently to inform the education and training sector.

The Spiritual Care-Giving Scale (SCGS), a self-reported scale, was developed by Tiew [21]. Although the SCGS is primarily designed to measure nursing students’ perspectives on spirituality or spiritual care, it has been used with nurses and is considered valid and reliable [22]. However, a critical question is whether this scale can be used with health providers from other cultural contexts, such as nurses in China. This study was designed to evaluate the psychometric properties of the SCGS in a Chinese-speaking nurse population and therefore had two main aims: 1) to translate the English version of the SCGS into Chinese and make cultural adjustments and 2) to evaluate the validity and reliability of the Chinese version (C-SCGS) for use with nurses from China. We hope our study will serve as a reference for the measurement, assessment and development of Chinese nurses’ spiritual care knowledge, perception, and skill.

## Methods

### Study design and participants

Nurses were recruited from three university-affiliated comprehensive hospitals, two cancer centers, one psychiatric hospital, and two traditional Chinese medicine hospitals to complete this cross-sectional study. Nurses who were reluctant to participate in the study were excluded. A convenience sample of 400 nurses was recruited, which was adequate for exploratory factor analysis (EFA) according to the guideline of Monte Carlo study decision on sample size [23].

Data were collected between March and April 2018. This study was approved by the Institute Review Board of College of Nursing, Jilin University (access number: 2018031102).

### Instruments

#### The self-designed general condition questionnaire

This form consisted of five questions about the participants’ age, gender, education, length of work experience, and department, which demonstrated that the participants recruited are a representative sample of different backgrounds.

#### The SCGS

The SCGS is a 35-item self-reporting measure created by Tiew and Creedy [21]. The scale has significant test-retest reliability (r = 0.811; *p* < 0.01). It includes five core factors: attributes of spiritual care (Cronbach’s alpha 0.926), spiritual perspectives (Cronbach’s alpha 0.896), definitions of spiritual care (Cronbach’s alpha 0.868), spiritual care attitudes (Cronbach’s alpha 0.879), and spiritual care values (Cronbach’s alpha 0.822). The SCGS revealed good psychometric properties.

#### The Chinese version of the spiritual care competency scale (C-SCCS)

The 27-item Spiritual Care Competency Scale (SCCS) developed by Leeuwen and colleagues [24] measures the competence of nurses in the provision of spiritual care to patients. The SCCS measures content in the same subject but in different dimensions and was therefore used to test the concurrent validity of the C-SCGS. The original SCCS consists of six factors with Cronbach’s alpha values from 0.71 to 0.82. The Chinese version, the C-SCCS, was translated and evaluated (using the same EFA method as for the translation and evaluation of the SCGS) by our study team with the permission of Dr. Leeuwen. It has three different components with good psychometric properties: the assessment, implementation, improvement, and professionalization of spiritual care (SCCS 1; Cronbach’s alpha = 0.934); personal and team support (SCCS 2: Cronbach’s alpha = 0.917); and attitude towards patient spirituality and communication (SCCS 3: Cronbach’s alpha = 0.855). The C-SCCS uses a five-point Likert rating response ranging from one (strongly disagree) to five (strongly agree).

#### Translation and adaptation procedures and psychometric testing

The English version of the SCGS was translated into Chinese according to Brislin’s translation model [25]. Permission to translate it was obtained from Dr. Tiew, the developer of the original SCGS. Phase I involved four steps. 1) Forward translation: two bilingual researchers interpreted the original SCGS into Chinese. After that, all members of the research team reviewed and discussed any incongruity in the two copies until consensus was reached. 2) Back-translation: the translated version was then back-translated blindly into English by two experts not working in the nursing field. One studied and worked in an English-speaking country for 7 years; the other has been teaching English for many years in a university in China. Then, the two back-translation versions were compared, verified, and revised by Tiew himself, from which a final Chinese translation was obtained. 3) Evaluation of translation equivalence: the translation validity index (TVI) [26, 27] was used to assess the translation equivalence of the versions. A total of five experts were recruited to compare the original English version of the SCGS and the Chinese version. The TVI assessment form was a 4-point Likert scale (1 = not relevant, 2 = needs major item modification to be equivalent, 3 = equivalent but needs minor modification, and 4 = equivalent). Every item was revised until a translation equivalence score of 4 was achieved. 4) Evaluation of content validity: an expert panel was asked to evaluate each item on a four-point Likert scale (from irrelevant to absolutely relevant) to determine the content validity of each item and to confirm whether the items were designed properly to create the constructs. The expert panel included one specialist in oncology (age, 48 years; working years, 29 years; position, ICU charge nurse; title, vice-high-level), one nurse in an intensive care unit (age, 35 years; working years, 10 years; position, nurse; title, intermediate grade), two nursing professors (one: age, 48 years; working years, 19 years; position, associate dean; title, associate professor, Ph.D.; the other: age, 63 years; working years, 46 years; title, professor, Ph.D.), and one advanced-practice nurse specializing in palliative care (age, 32 years; working years, 10 years; position, head nurse; title, charge nurse). The items were evaluated individually. Ambiguous and/or complex terms were removed or rephrased until no changes to the Chinese translation were deemed necessary. We made some cultural adjustments to the expression of certain items according to the experts’ advice after two rounds of consultation.Three items on religion were considered inappropriate by some experts. However, although only a small number of Chinese nurses have religious beliefs, the reciprocal relationship between nurses’ religious beliefs and spiritual care seems obvious, and therefore, the three items were retained.

Phase II consisted of two steps. 1) The revised version of the SCGS was pilot tested to evaluate whether the C-SCGS was easy to understand and answer in three Jinlin University affiliated teaching hospitals with a convenience sample of 17 nurses. 2) The psychometric properties of the C-SCGS were evaluated, including item analysis, construct validity, concurrent validity, internal consistency reliability, and split-half reliability. The construct validity of the C-SCGS was determined by performing the principal axis factoring extraction method with Promax with Kaiser normalization rotation. The concurrent validity refers to the comparison of the results of a test using the targeted instrument with those of another effective test using another valid measuring method at the same time by adopting a quantitative method of calculating the correlation coefficient. In this study, the Pearson correlation coefficients of the C-SCGS and the Chinese version of the Spiritual Care Competency Scale (C-SCCS) were calculated to assess the concurrent validity of the C-SCGS. To verify the quality of the component structure, we conducted a confirmatory factor analysis (CFA) based on an additional data sample obtained from 351 nurses. (In this study, a total of 707 data points were collected and divided into two samples by a computerized random method using *SPSS 17.0*. One sample, including the information from 355 nurses, was used for EFA, and the other, including the information from 351 nurses, was used for CFA. Although a convenient sampling method was adopted for sample selection, stratification sampling was used as far as possible, taking into account the multiple departments of various types and levels of hospitals, to obtain a representative sample reflecting the topic of this study. See Table 1). We also tested the internal consistency and stability of the C-SCGS by using Cronbach’s alpha coefficient and the Guttman split-half coefficient, respectively.

**Table 1:** Social and demographic information of the participants (*n* = 355)

Descriptive characteristics	Frequency (n)	Percentage (%)
Gender		
Male	17	4.8
Female	338	95.2
Age, years		
≥18	54	15.2
≥26	130	36.6
≥31	129	36.3
≥41	36	10.1
≥51	6	1.7
Marital status		
Unmarried	96	27.0
Married	253	71.3
Divorced or widowed	6	1.7
Education		
Secondary vocational schools	3	.8
Junior college	72	20.3
Undergraduate	250	70.4
Postgraduate or above	30	8.5
Income(¥/month)		
<5000	193	54.4
≥5000	162	45.6
Working years (M ± SD)	10.3	8.7
Type of hospital		
Comprehensive hospital	150	42.3
Tumor hospital	35	9.9
Psychiatric hospital	48	13.5
Traditional Chinese medicine (TCM) hospital	116	32.7
Marital and child service care center	2	.6
Others	4	1.1
Department		
Internal medicine	126	35.5
Surgical	53	14.9
Pediatric	13	3.7
Obstetrics and gynecology	43	12.1
Emergency	11	3.1
ICU	12	3.4
The operating room	8	2.3
Outpatient service	11	3.1
Psychiatric	42	11.8
Others	36	10.1
Professional title		
Primary nurse	84	23.7
Nurse practitioner	148	41.7
Nurse-in-charge	112	31.5
Deputy director nurse	11	3.1

### Data collection

A professional platform called SO JUMP was used for the data collection [28]. First, the content of the questionnaire was entered into a computer. Then, the questionnaire was sent to individual nurses through WeChat (a total of 17 nurses) and to 4 WeChat chat groups with relatively fixed numbers of nurses (group 1, 52; group 2, 65; group 3, 60; and group 4, 206) over WhatsApp (three of these chat groups were established as part of this research, and one has been used previously for the continuing education of a nurse team). Before answering the questionnaires, all participants were asked to sign a written consent form.

### Statistical analysis

Statistical analyses were performed using SPSS 17.0 for Windows. Categorical variables were expressed as frequencies and percentages. Continuous variables were presented as the mean ± standard deviation (SD) if the distribution was normal. Every subscale’s internal consistency and homogeneity was assessed by Cronbach’s alpha. Concurrent validity was assessed by the Pearson correlation coefficient between SCGS and SCCS. Item analysis was performed using the following analyses: (a) item analysis, (b) corrected item-total correlation, (c) factor loading, (d) Cronbach’s alpha if an item was deleted, (e) extreme group comparison, and (f) communities. Items that had a criterial value (CR) < 3.0, a corrected item-total correlation < 0.30, factor loading < 0.40, community < 0.20 and whose deletion caused an increase of 0.5 or more in the alpha coefficient for the overall scale were excluded.

An EFA was performed by exploring the main components in the correlation matrix of every item, with a Promax with Kaiser normalization rotation and the Kaiser criterion to test the construct validity of the SCGS if the correlation coefficient between the factors was greater than 0.3. Prior to performing EFA, Kaiser–Meyer–Olkin (KMO) and Bartlett’s sphericity test were used to test the sampling adequacy and the suitability of the data for factor analysis. The criterion for factor extraction was an eigenvalue > 1.0 since these values could explain a higher percentage of the total variability and a factor loading of > 0.40. If the results of the standard EFA were not consistent with the original theoretical model of the SCGS, an alternative methodological consideration was to perform separate EFA for each SCGS subscale to evaluate the subscale dimensionality [29, 30]. To contrast differences in the mean values of a quantitative variable, Student’s t-test (in a continuous scale of two independent populations) or the F test was used (in a continuous scale of three or more independent populations). A *p* value < 0.05 was accepted as statistically significant.

AMOS version 20.0 was used to perform the CFA to further evaluate the validity of the C-SCGS.

## Results

### Sample characteristics

A total of 356 (out of a possible 400) nurses completed the survey, providing a response rate of 88.75%. The majority of nurses were female (*n* = 338, 95.2%), married (71.3%), and had completed undergraduate education (70.4%). The average length of employment was 10.3 years. Sample characteristics are presented in Table 1.

### Psychometric analyses

To assess face validity, the C-SCGS was given to 20 nurses (having more than 5 years of working experience; being familiar with the concepts of spirituality and spiritual care; working in the ICU, oncology or palliative care unit) to understand how they perceived and interpreted the items by means of a purposive sampling strategy. The participants reported that the wording of the C-SCGS was clear and that they had little difficulty understanding it.

During the homogeneity analyses for each item, all 35 items of the SCGS were retained. Although the communality coefficient of item 1 (Q1) of the C-SCGS was lower than 0.20 (0.172) and the corrected item-total correlation score was 0.390 (An additional table file shows this in more detail [see Additional file 1]), the results of expert consultation showed that four out of five experts believed the item had clinical significance. In addition, other indicators of this item meet the standards, so the experts recommended keeping this item. The internal consistency analysis of the 35-item C-SCGS showed that the Item–total correlations ranged from 0.427 to 0.829; CR values were all greater than 3.0; and all corrected item-total correlations ranged from 0.390 to 0.817, indicating moderate to strong correlation (Table 2). No item deletion would have improved the Cronbach’s alpha value of the scale.

**Table 2:** Results of the Exploratory factor analysis of 34-items^a^ C-SCGS (n=355)

The structure matrix of the promax oblique rotation axis					The pattern matrix of the promax oblique rotation axis					C^2^
Items	Factor 1	Factor 2	Factor 3	Factor 4	Items	Factor 1	Factor 2	Factor 3	Factor 4	
B28	.820	.423	.475	.629	B28	.873	-.135	-.125	.145	.425
B27	.796	.414	.513	.635	B29	.796	.079	-.063	-.061	.533
B23	.772	.512	.537	.638	B27	.787	-.130	-.040	.153	.597
B25	.768	.476	.506	.571	B32	.786	.070	.053	-.150	.571
B24	.765	.484	.593	.622	B25	.744	.023	.041	-.022	.482
B29	.759	.500	.429	.529	B34	.706	-.143	.089	.043	.374
B31	.757	.503	.545	.558	B30	.698	.105	-.119	.074	.530
B32	.750	.481	.472	.508	B31	.689	.087	.147	-.103	.592
B30	.741	.520	.416	.567	B23	.634	.052	.035	.115	.379
B35	.725	.398	.537	.626	B24	.621	.027	.170	.027	.558
B34	.708	.342	.508	.548	B35	.603	-.097	.060	.192	.527
B26	.688	.535	.506	.513	B26	.555	.207	.161	-.126	.551
B16	.637	.612	.455	.622	B22	.389	.268	-.174	.252	.495
B22	.627	.575	.355	.569	B21	.111	.828	-.071	-.262	.477
B21	.371	.720	.146	.234	B20	-.001	.701	-.108	.051	.449
B20	.391	.686	.206	.367	B15	-.149	.643	.118	.101	.515
B18	.611	.664	.445	.570	B17	-.003	.617	.014	.034	.411
B15	.388	.656	.350	.433	B6	-.169	.485	.112	.252	.521
B19	.628	.655	.448	.527	B18	.226	.440	.035	.132	.517
B17	.403	.640	.282	.387	B19	.325	.434	.077	-.010	.477
B6	.381	.569	.375	.477	B16	.266	.312	-.024	.267	.566
B3	.569	.370	.760	.624	B1	-.121	.008	.740	-.032	.480
B4	.548	.367	.749	.578	B2	.129	-.103	.731	-.069	.608
B2	.477	.227	.723	.480	B4	.087	.033	.654	.038	.606
B5	.487	.390	.681	.540	B3	.080	.002	.616	.133	.591
B1	.327	.211	.643	.399	B5	-.008	.124	.593	.062	.509
B8	.627	.458	.547	.764	B8	.135	.008	-.006	.663	.649
B12	.588	.393	.577	.734	B12	.094	-.053	.105	.622	.691
B11	.608	.414	.522	.717	B11	.183	-.031	.001	.598	.584
B10	.576	.517	.619	.714	B13	.162	.181	-.130	.538	.565
B7	.558	.274	.639	.672	B9	-.186	.288	.038	.522	.588
B14	.581	.351	.536	.672	B14	.202	-.093	.086	.514	.572
B13	.587	.527	.420	.668	B10	-.031	.177	.239	.471	.520
B9	.398	.483	.400	.572	B7	.135	-.189	.303	.466	.551

KMO = 0.951, Bartlett (*p* value) ^2^=7632.213, df=561, *p* = 0.000; Extraction Method: Principal Axis Factoring; Rotation Method: Promax with Kaiser Normalization

^a^Excluded:B33 Spiritual care requires the nurse to be empathetic towards the patient 心灵关怀要求护士对患者富有同情心

The Chinese version of the Spiritual Care-Giving Rating Scale (C-SCGS): Attributes for Spiritual Care (Factor 1), Defining Spirituality and Spiritual Care (Factor 2), Spirituality Perspectives (Factor 3), Spirituality and Spiritual Care Values (Factor 4). Item 16 entried in factor 2

**Fig. 1:**
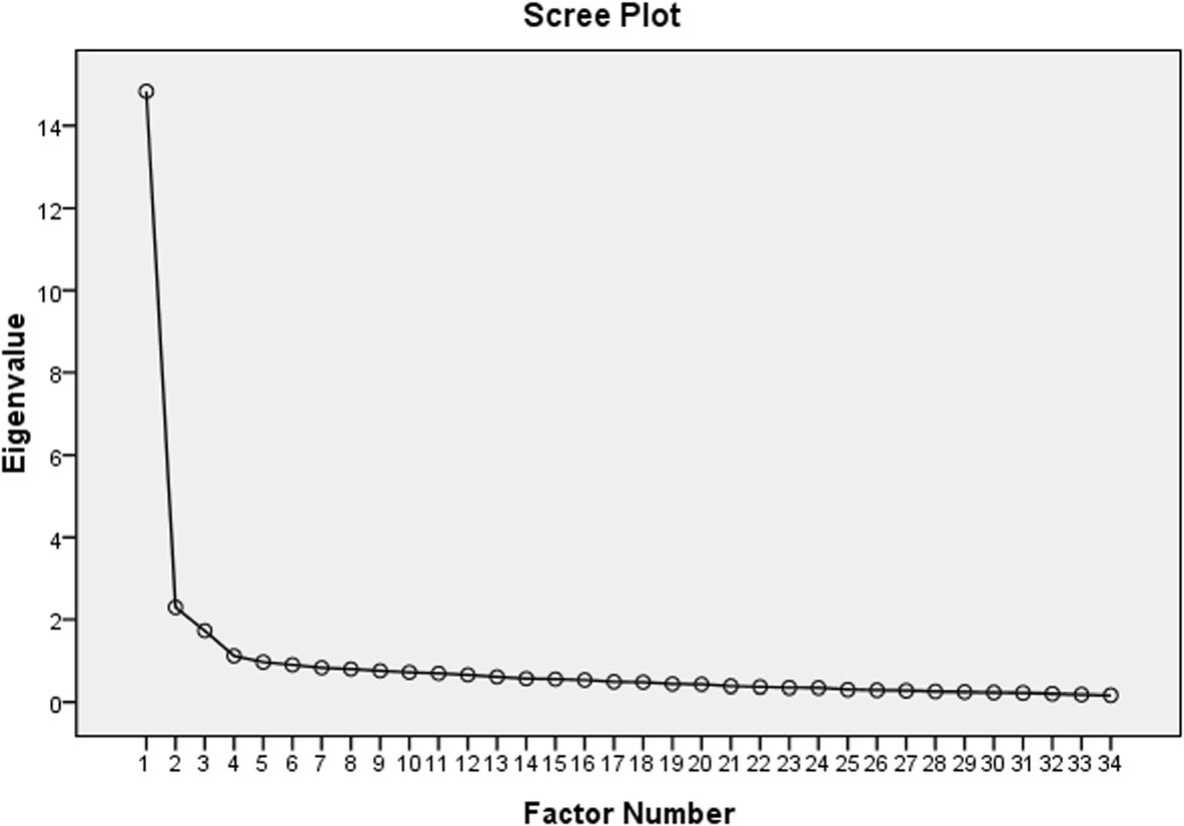
Scree plot

To test the validity and reliability of the questionnaire, a factorial analysis was performed that showed the presence of an underlying structure composed of four factors (see Fig. 1. Scree plot), according to the Kaiser-Meyer-Olkin criteria. Bartlett’s sphericity test was also performed. The correlation coefficients of the factors were all greater than 0.30 (Table 3). Therefore, this factor analysis was suitable for Promax with Kaiser normalization rotation. Item 33 was excluded because Factor 5 of the factorial analysis included only one item Q33 (An additional table file shows this in more detail [see Additional file 2]). The final questionnaire contained 34 items with a range of possible values between 134 and 210 points. The factors found in the factorial analysis accounted for 53.116% of the variance, and every factorial item had a value> 0.40. The homogeneity tests for each of the four factors demonstrated a Cronbach’s alpha value above 0.700, and none of them were removed. After Promax with Kaiser normalization rotation, Factor 1 included thirteen items related to ‘attributes of spiritual care’ (items 22, 23, 24, 25, 26, 27, 28, 29, 30, 31, 32, 34, and 35), Factor 2 included eight items related to ‘definitions of spirituality and spiritual care’ (items 6, 15, 16, 17, 18, 19, 20, and 21), Factor 3 included five items related to ‘spiritual perspectives’ (items 1, 2, 3, 4, and 5), and Factor 4 contained eight items related to ‘spirituality and spiritual care values’ (items 7, 8, 9, 10, 11, 12, 13, and 14). The structure matrix showed that the factor loadings on Factor 1 (0.637) and Factor 2 (0.612) were similar, but the pattern matrix showed that item 16 was more important for Factor 2. Therefore, item 16 was included in Factor 2. (The items included in each of the four factors are shown in Table 4). For the four subscales of the C-SCGS, the corresponding Cronbach’s alpha coefficients were 0.941, 0.852, 0.836, and 0.866, respectively. Table 4 shows the adjusted item-total correlations, Cronbach’s alphas if an item was deleted, means, standard deviations, and cumulative interpretation of variance. The Guttman split-half coefficient of C-SCGS was 0.893, indicating adequate reliability.

**Table 3:** The factor correlation matrix

Factor Correlation Matrix				
Factor	1	2	3	4
1	1.000	.603	.632	.741
2	.603	1.000	.399	.560
3	.632	.399	1.000	.699
4	.741	.560	.699	1.000

Extraction Method: Principal Axis Factoring

Rotation Method: Promax with Kaiser Normalization.

**Table 4:** The results of factor analysis of the 34-item Spiritual Care-Giving Chinese version (C-SCGS) (n=355)

Items	Corrected item-factor correlation	Cronbach's α if item deleted	Average Mean	SD
Factor 1^b^ Attributes for Spiritual Care 维度1:灵性照护特点 (Cronbach’s α = 0.941; Guttman Split-Half coefficient=0.902)			5.01	0.49
B22 I am comfortable providing spiritual care to patients. 我舒心地为患者提供心灵关怀	.605	.941	4.87	.821
B23 Nurses provide spiritual care by respecting the dignity of patients. 护士通过尊重患者的尊严为其提供心灵关怀	.760	.935	5.03	.694
B24 Spiritual care should take into account of what patients think about spirituality 心灵关怀应考虑到患者的心灵理念	.750	.935	5.03	.621
B25 Nurses who are spiritual aware are more likely to provide spiritual care. 具有心灵意识的护士更有可能提供心灵照护	.748	.935	5.04	.678
B26 Spiritual care requires awareness of one's spirituality 心灵关怀需意识到自身的心灵世界	.666	.938	5.00	.703
B27 Spiritual care should be instilled throughout a nursing education programme 心灵关怀的理念应融入护理教育课程	.760	.935	5.05	.700
B28 Spiritual care should be positively reinforced in nursing practice. 心灵关怀应在护理实践中得到该积极加强	.782	.934	5.08	.698
B29 The ability to provide spiritual care develops through experience. 提供心灵关怀的能力通过经历/体验得以发展	.730	.936	4.98	.701
B30 Spiritual care is important because it gives patient hope 心灵关怀因给予患者希望而有价值	.718	.936	4.98	.729
B31 Spirituality is influenced by individual’s life experiences. 灵性(心灵)受个人生活经历的影响	.740	.935	5.00	.674
B32 Spirituality helps when facing life’s difficulties and problems. 灵性(心灵)助力/帮助面对生活的困难和问题	.721	.936	4.94	.728
B34 A trusting nurse-patient relationship is needed to provide spiritual care 提供心灵关怀需基于信任的护患关系	.682	.937	5.13	.690
B35 A team approach is important for spiritual care 团队的方式对心灵关怀很重要	.707	.936	5.04	.677
Factor 2^b^ Defining Spirituality and Spiritual Care 维度2:灵性与灵性照护定义(Cronbach’s α= 0.852; Guttman Split-Half coefficient=0.853)			4.73	.89
B6 Spirituality is about finding meaning in the good and bad events of life 灵性是指寻找生活中好坏事件的意义	.527	.843	4.57	1.064
B15 Spiritual care is respecting a patient’s religious or personal beliefs 心灵关怀即尊重患者的宗教或个人信仰	.609	.832	4.87	.941
B16 Sensitivity and intuition help the nurse to provide spiritual care. 敏感性和直觉助力护士提供心灵关怀	.588	.837	4.94	.722
B17 Being with a patient is a form of spiritual care. 心灵关怀的形式之一是与患者在一起	.611	.832	4.59	1.034
B18 Nurses provide spiritual care by respecting the religious and cultural beliefs of patients. 护士通过尊重患者的宗教和文化信仰为其提供心灵关怀	.649	.830	4.90	.756
B19 Nurses provide spiritual care by Giving patients time to discuss and explore their fears, anxieties and troubles 护士给予患者足够的时间谈论和探究其恐惧、焦虑和烦恼为其提供心灵关怀	.620	.832	4.90	.795
B20 Spiritual care enables the patient to find meaning and purpose in their illness 心灵关怀使患者找到其患病的意义和目的	.615	.831	4.61	1.053
B21 Spiritual care includes support to help patients observe their religious beliefs 心灵关怀即支持或帮助患者保持其宗教信仰	.593	.835	4.47	1.105
Factor 3^b^ Spiritual Perspectives 维度3:灵性认知 (Cronbach’s α = 0.836; Guttman Split-Half coefficient=0.759)			5.09	0.52
B1 Everyone has spirituality. 每个人都有灵性(心灵)	.570	.825	5.12	.785
B2 Spirituality is an important aspect of human beings. 灵性是人类的一个重要方面	.673	.793	5.15	.719
B3 Spirituality is part of a unifying force which enables individuals to be at peace 灵性是能使人平和/安宁、和睦的达成一致的力量部分	.685	.790	5.01	.740
B4 Spirituality is an expression of one’s inner feelings that affect behaviour. 灵性是一种影响人的行为的内在情感表达	.672	.793	5.03	.720
B5 Spirituality is part of our inner being. 灵性是我们内心的一部分	.602	.814	5.12	.622
Factor 4^b^ Spirituality and Spiritual Care Values 维度4: 灵性与灵性照护价值 (Cronbach’s α= 0.866; Guttman Split-Half coefficient=0.849)				
B7 Spiritual well-being is important for one’s emotional well-being 心灵的幸福对个体的情感健康很重要	.622	.850	5.25	.699
B8 Spirituality drives individuals to search for answers about meaning and purpose in life. 灵性驱使个人寻找生活的意义及其目的的答案	.699	.842	5.01	.715
B9 Without spirituality, a person is not considered whole. 没有灵性,就不是个完整的人	.534	.876	4.47	1.175
B10 Spiritual needs are met by connecting oneself with other people, higher power or nature. 灵性的需求通过自己与他人、更大能量或自然界的联系得到满足	.677	.843	4.87	.783
B11 Spiritual care is an integral component of holistic nursing care 心灵关怀是整体护理的重要组成	.655	.847	5.10	.679
B12 Spiritual care is more than religious care. 心灵关怀不只是宗教的关怀	.681	.846	5.11	.635
B13 Nursing care, when performed well, is itself, spiritual care. 良好的护理本身就是心灵关怀	.628	.849	5.01	.828
B14 Spiritual care is a process and not a one- time event or activity. 心灵关怀是个过程, 而不是一次性事件或活动	.633	.850	5.19	.646
Cumulative interpretation of variance%				53.116

*SD* Standard deviation

^b^ Spearman-Brown Coefficient=0.908; 0.853; 0.811; 0.856; respectively

Regarding the concurrent validity of C-SCGS, the correlations of overall C-SCGS and its four factors listed above with the overall SCCS were 0.534, 0.515, 0.490, 0.310, and 0.439, respectively (*p* < 0.01; see Table 5).

**Table 5:** Cronbach’s alpha and Pearson’s Product-Moment Correlation between the Two Scales (SCGS & C-SCCS)

Measures	C-SCGS	Factor 1	Factor 2	Factor 3	Factor4	C-SCCS	C-SCCS 1	C-SCCS 2	C-SCCS 3
C-SCGS									
Factor 1	0.924**	α=0.941							
Factor 2	0.829**	0.661**	α=0.852						
Factor 3	0.741**	0.618**	0.451**	α=0.836					
Factor4	0.891**	0.763**	0.634**	0.675**	α=0.866				
C-SCCS	0.534**	0.515**	0.490**	0.310**	0.439**				
C-SCCS 1	0.519^**^	0.497^**^	0.464^**^	0.307^**^	0.441^**^	0.441^**^	α=0.934		
C-SCCS 2	0.342^**^	0.309^**^	0.404^**^	0.134^*^	0.248^**^	0.248^**^	0.731^**^	α=0.917	
C-SCCS 3	0.526^**^	0.550^**^	0.359^**^	0.402^**^	0.452^**^	0.452^**^	0.510^**^	0.392**	α=0.855

Pearson’s correlation coefficient test was used, two-tailed. Cronbach’s alpha on the diagonal in parenthesis. Spiritual Care-Giving Rating Scale (C-SCGS): Attributes for Spiritual Care (Factor 1), Defining Spirituality and Spiritual Care (Factor 2), Spirituality Perspectives (Factor 3), Sprituality and Spiritual Care Values (Factor 4). Spiritual Care Competency Scale (C-SCCS): Assessment, implementation, professionalization and quality improvement of spiritual care (C-SCCS 1), Personal and team support (C-SCCS 2), Attitude towards patient spirituality and communication (C-SCCS 3)

* *p* < 0.05; ** p < 0.01

The four-factor model was also chosen to conduct confirmatory factor analysis using another data set from a sample of 351 nurses. The present model provides an acceptable fit to the data (CMIN/DF = 2.14; root mean square error of approximation, RMSEA = 0.06; goodness-of-fit index, GFI = 0.88; incremental fit index, IFI = 0.92; adjusted goodness-of-fit index, TLI = 0.91; comparative fit index, CFI = 0.92; An additional figure file shows this in more detail [see Additional file 3].)

**Fig. 2:**
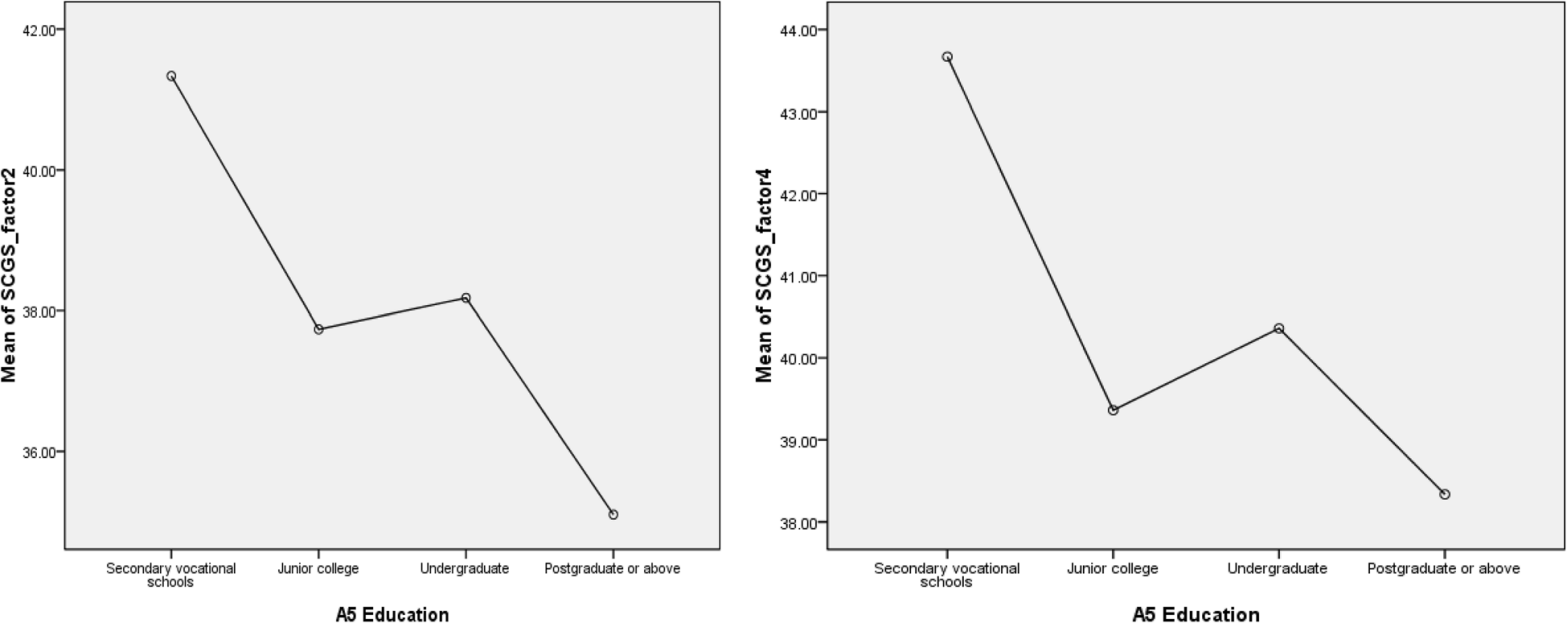
Means Plots of SCGS_factor 2 and factor 4

Table 6 shows the association between nurses’ demographic variables and the four factors of the C-SCGS. We found significant associations with Factor 2, ‘Defining Spirituality and Spiritual Care’ (F = 3.540, *p* = 0.015), and Factor 4, ‘Spirituality and Spiritual Care Values’ (F = 3.069, *p* = 0.028). The nurses with secondary vocational schooling and undergraduate-level education appeared to score higher in perceptions of spirituality and spiritual care. The post hoc analysis (also see Fig. 2. Means Plots of SCGS_factor 2 and factor 4) showed a significantly higher proportion of undergraduate nurses who possessed more positive perspectives on ‘Defining Spirituality and Spiritual Care’ and ‘Spirituality and Spiritual Care Values’ than nurses with graduate-level education and above (mean difference (I-J), 3.08; std. error, 1.01; *p* = 0.03 for undergraduate nurses; mean difference (I-J): 2.03; std. error: 0.87; p = 0.02 for graduate nurses). Neither gender, age nor income were associated with nurses’ perceptions of spirituality and spiritual care.

**Table 6:** Association between the Chinese version of the Spiritual Care-Giving Scale and personal issues

Test Variable	Groups	Frequency (n)	Total (M±SD)	Factor 1 (M±SD)	Factor2 (M±SD)	Factor 3 (M±SD)	Factor 4 (M±SD)
Gender	Male	17	162.29±19.83	63.41±7.27	36.41±6.10	24.29±3.18	38.18±6.26
	Female	338	168.79±16.76	65.27±6.97	37.93±5.25	25.49±2.77	40.11±4.41
t value			-1.545	-1.071	-1.153	-1.719	-1.256
P value			.123	.285	.250	.087	.226
Age, years	≥18	54	168.70±16.48	65.41±6.59	38.22±4.96	25.43±2.66	39.65±4.46
	≥26	130	167.53±16.31	64.83±6.97	37.72±5.01	25.24±2.79	39.74±4.69
	≥31	129	168.75±17.24	65.28±7.15	37.90±5.48	25.40±2.85	40.17±4.31
	≥41	36	170.61±18.25	65.86±6.83	37.56±6.21	26.31±2.69	40.89±4.76
	≥51	6	168.33±24.22	64.50±9.65	38.33±6.02	24.83±3.49	40.67±5.47
F value			.253	.200	.127	1.108	.612
P value			.908	.938	.973	.353	.654
Education	Secondary vocational schools (A)	3	182.33±34.96	70.00±13.86	41.33±9.87	27.33±4.62	43.67±6.66
	Junior college (B)	72	167.36±16.23	65.17±6.45	37.74±4.87	25.11±2.53	39.36±4.67
	Undergraduate (C)	250	169.18±16.80	65.13±6.96	38.18±5.31	25.50±2.78	40.36±4.28
	Postgraduate or above (D)	30	163.93±17.46	65.13±7.82	35.10±5.02	25.37±3.35	38.33±4.54
F vaue			1.640	.479	3.540	.838	3.069
P value			.180	.697	.015*	.474	.028*
Post Hoc Tests	Scheffe’s method				C>D^a^		
	Tukey HSD				C>D^b^		
	LSD method				B>C^c^; C>D^d^;		C>D^e^
Income(¥/month)	<5000	193	169.31±17.44	65.46±7.14	38.33±5.32	25.33±2.80	40.19±4.45
	≥5000	162	167.49±16.34	64.85±6.80	37.29±5.22	25.55±2.79	39.81±4.62
t value			1.003	.827	.827	-.748	.783
P value			.316	.409	.065	.455	.434

* p < 0.05; *LSD* Least significant difference, *HSD* Honestly significant difference

^a^ Mean Difference (I-J), 3.08*; Std. Error, 1.01; P=.03

^b^ Mean Difference (I-J): 3.08*; Std. Error: 1.01; P=.01

^c^ Mean Difference (I-J): 2.64*; Std. Error: 1.14; P=.02

^d^ Mean Difference (I-J): 3.08*; Std. Error: 1.01; P=.00

^e^ Mean Difference (I-J): 2.03*; Std. Error: .87; P=.02

## Discussion

It is important to find a valid and reliable measurement to identify nurses’ current perspectives on spiritual care and spirituality to inform the education and training sector, especially in China. The main aim of this study was to translate the SCGS into Chinese and examine its reliability and validity. The participants of this study were recruited from eight different types and levels of hospitals and various departments. Therefore, the results represented nurses with diverse backgrounds.

The main finding of this article is that, after excluding one item of the current SCGS from the homogeneity and factorial analyses, this tool is useful to identify nurses’ perspectives on spirituality and spiritual care in nursing practice. Thus, in the final version of the C-SCGS, high scores are strongly correlated with spiritual care-giving level. Additionally, spiritual care perception levels were associated with education in this cohort of nurses.

Compared with the original English version of the SCGS, the C-SCGS performed well in four dimensions. An EFA determined 34 items categorized under four factors, which explained 53.116% of the total variance of every dimension. All items had a factor loading of 0.60 or higher, which was considered ideal. However, these results had minor differences from those reported in studies conducted by Tiew et al. [21], who performed an EFA with the whole scale including 35 items and obtained five common factors. We conducted a factor analysis for our samples using the same method (principal component analysis, PCA), but we found that the content of the items contained in the factors was quite different from the original theoretical structure. Similar to the results of the original SCGS with five factors with r = 0.581–0.765, all factors in the present scale were also significantly and moderately correlated (r = 0.399 to r = 0.741; *p* < 0.01) with each other. Thus, we used the principal axis factoring extraction method using the Promax with Kaiser normalization rotation method and found that the fifth factor contained only one item. After item 33 was deleted, the factor analysis was rerun, and a four-factor model was obtained.

The 34-item C-SCGS demonstrated acceptable concurrent validity. A statistically significant correlation (both scales measured similar subjects regarding spirituality and spiritual care, although one assesses perceptions of spirituality and spiritual care and the other evaluates the level of competence of spiritual care) was found between C-SCGS and C-SCCS (r = 0.534, *P* < 0.01; generally, 0.4 to 0.7 is considered to be moderately correlated), which revealed that the C-SCGS performed with acceptable concurrent validity when assessed against the C-SCCS. Therefore, to some extent, this scale was sensitive enough to evaluate similar features to those described by the C-SCCS.

The Cronbach’s alpha of the four factors, with values of 0.941, 0.852, 0.836, and 0.866, showed good internal consistency; the correlation of 0.893 of the split-half internal consistency test also suggested the sound reliability of C-SCGS. The results of this study were consistent with the results for the original English version, in which Cronbach’s alpha ranged from 0.811 to 0.90 [21]. The results were also consistent with a later test in nurses in Singapore in which a Cronbach’s alpha coefficient of 0.97 was obtained [22].

Concerning the translation of the C-SCGS, most items had culturally equivalent terms in Chinese. Therefore, we were able to translate the questionnaire without extensive adaptation. There were two exceptions. One was the concept of “spiritual.” Dr. Leeuwen was consulted on the translation and modification of some of the expressions. The other exception was that, to adapt to the cultural background, we used the Chinese word “心灵” as an equivalent to the word “灵性” (spirituality) in some sentence expressions because some experts and clinical nurses suggested that the direct translation of the word spirituality is not easy to understand. We consulted Dr. Tiew, the developer of SCGS, who is familiar with both English and Chinese, and obtained her approval about this adaptation. It should also be noted that some experts suggest deleting religion-related content from the C-SCGS because most nurses in China are nonreligious. However, given that the research team found a certain relationship between religion and spiritual cognition when they read the literature, and indeed some Chinese nurses believe in Christianity and Buddhism, the scale retained the items related to religion.

The number of items to be retained at each subscale was determined, and the factors were given similar labels to those provided by the original author: attributes of spiritual care (Factor 1); definitions of spirituality and spiritual care (Factor 2); spirituality perspectives (Factor 3); and spirituality and spiritual care values (Factor 4). The Chinese version of the Spiritual Care-Giving Scale can be seen in the supplementary table in Additional file 4.

This study has several shortcomings. First, the sample of nurses was mainly from the Henan and Jilin provinces of China and was obtained using a convenience sampling method; therefore, the findings may not represent the opinions of all nurses in China. Second, there is the possibility of a social desirability bias in the responses. The nurses may have chosen responses that were consistent with their leaders’ expectations even though their participation was voluntary and their anonymity ensured. This possibility is due to the special cultural and systemic background. In most departments in most hospitals in China, leaders have authority over employee bonuses and work arrangements. Additionally, leaders always hope for their organizations to have a positive image. As a result, employees may subconsciously conform to the expectations of their leaders. Third, the use of the online-based questionnaire format instead of the original paper-based SCGS could lead to differences in validity between the online and paper forms. The use of an online questionnaire could also influence on the responses due to unfamiliarity with online questionnaires and potential errors in answering using a mobile device.

The results have certain implications for future research. The C-SCCS was used to evaluate the concurrent validity of the C-SCGS because the C-SCCS measures the spiritual care competency of student nurses. There was a moderately strong relationship between the C-SCGS and C-SCCS. Future studies may attempt to establish a structural equation model (SEM) to further analyze the factors influencing nurses’ spiritual care perceptions and abilities and their relationships. The findings of the current study provide further support for the validity and reliability of the SCGS for the measurement of nurses’ awareness, knowledges, attitudes, and perspectives on spiritual care and spirituality. However, the C-SCGS CFA test supported the results of the fitting model after modification (see Fig. 2). This observation may be partly explained by the relatively small sample size, as large samples always show better RMSEA [31]. It may also means that the model has multiple collinearity due to cross loading and needs further modification. Future research should enlarge the sample size and try to use exploratory structural equation modeling (ESEM), which integrates features of CFA, EFA) and SEM in a single framework to overcome certain limits of CFA [32, 33].

This study has implications for clinical nursing. First, given the evidence for the advantages of recognizing nurses’ perspectives of spirituality and spiritual care, in recent years, studies assessing this subject have increased [18, 34–36]. Although some of them show that spiritual care can improve patients’ and all people’s health outcomes, the topic is not given adequate attention in nursing practice. One of the main barriers may be limitations of nurses’ understanding of spirituality and spiritual care, which is essential for best practice. To provide spiritual care, nurses need to be knowledgeable regarding the topics of spirituality and spiritual care and their own perceptions and attitudes towards this issue. Identifying these issues using a valid instrument proven by the current study will allow nurses to explore available resources to assist in improving their knowledge and ability to provide spiritual care and meet patients’ spiritual needs. Second, although the SCGS was first developed as an instrument to assess student nurses’ spirituality and spiritual care perspectives, it was found to be a valid and reliable multidimensional tool for use in Chinese clinical nursing staff with a multicultural background. Findings from the C-SCGS would assist clinical nursing managers in evaluating staff members’ understanding of spirituality and spiritual care and their perspectives on these topics. Identifying problems in these areas will allow managers to formulate strategies to empower nurses through education, provide them with spiritual care skills for optimal practice, and assist them in improving care quality.

Importantly, our results also revealed that nurses with lower levels of education (junior college and undergraduate) scored higher on the C-SCGS. Additionally, undergraduate nurses reported, statistically significantly higher level perceptions of spirituality and spiritual care than nurses with graduate-level education or above. This difference may be because of the small sample of nurses with graduate and above education. However, there might be other reasons which should be explored with greater attention.

## Conclusions

The results of this study showed that the 34-item C-SCGS has satisfactory concurrent validity, construct validity, and internal consistency. It was found to be a potentially helpful instrument to measure mainland Chinese nurses’ perceptions regarding spirituality and spiritual care. Education was significantly associated with the scores in the C-SCGS and therefore with different perceptions of spirituality and spiritual care levels. The details of this association and its reasons need to be analyzed further. Future research should try to use ESEM to overcome some of the limits of CFA and to further verify the validation of the C-SCGS used in this study, recruit a larger sample that is more representative of the Chinese nursing population, translate the C-SCGS into other native dialects, or apply it in other settings such as palliative care.

## Additional files

Additional file 1: **Table S1.** Internal consistency of 35-items C-SCGS (*n* = 355). (DOCX 20 kb)

Additional file 2: **Table S2.** The Structure Matrix of the 35-items C-SCGS. (DOCX 24 kb)

Additional file 3: **Figure S1.** Results of the confirmatory factor analysis. (DOCX 253 kb)

Additional file 4: The Chinese version of the Spiritual Care-Giving Scale. (DOCX 20 kb)

## Abbreviations

CFA: Confirmatory factor analysis
CR: The criterial value
C-SCCS: The Chinese version of the Spiritual Care Competency Scale
C-SCGS: The Chinese version of the Spiritual Care-Giving Scale
CVI: The content validity of item
EFA: Exploratory factor analysis
ESEM: Exploratory structural equation modeling
KMO: Kaiser–Meyer–Olkin
SCGS: The Spiritual Care-Giving Scale
SEM: Structural Equation Model
SSCRS: The Spirituality and Spiritual Care Rating Scale
TVI: The Translation Validity Index
