# Supplementary material for: Psychometric properties of the Chinese version of the spiritual care-giving scale (C-SCGS) in nursing practice
Source: BMC Med Res Methodol. 2019 Jan 23;19:21. doi: 10.1186/s12874-019-0662-7 (PMC6343288; doi:10.1186/s12874-019-0662-7)
Supplement: Supplementary file 1 — Table S1. Internal consistency of 35-items C-SCGS (n = 355). (DOCX 20 kb) [file 12874_2019_662_MOESM1_ESM.docx]

**Table S1** Internal consistency of 35-items C-SCGS (n＝355)

| **Items** | **Mean ± SD** | **Critical value** | **Item-total correlation** | **Adjusted item-total correlation** | **Cronbach^'^s α if item deleted** | **C^2^** | **Factor loading** |
| --- | --- | --- | --- | --- | --- | --- | --- |
| B1 | 5.05 ± 0.85 | 5.999** | .427** | .390 | .966 | .172 | .415 |
| B2 | 5.14 ± 0.76 | 9.825** | .569** | .541 | .965 | .322 | .567 |
| B3 | 5.11 ± 0.68 | 13.310** | .672** | .652 | .965 | .452 | .672 |
| B4 | 5.09 ± 0.75 | 12.518** | .674** | .651 | .964 | .450 | .671 |
| B5 | 5.18 ± 0.66 | 12.268** | .686** | .667 | .964 | .475 | .689 |
| B6 | 4.78 ± 0.97 | 8.399** | .523** | .485 | .966 | .240 | .490 |
| B7 | 5.29 ± 0.68 | 11.536** | .646** | .624 | .965 | .422 | .650 |
| B8 | 5.03 ± 0.77 | 16.450** | .749** | .730 | .964 | .550 | .741 |
| B9 | 4.74 ± 1.12 | 9.428** | .526** | .482 | .966 | .247 | .497 |
| B10 | 4.93 ± 0.83 | 13.426** | .712** | .689 | .964 | .494 | .703 |
| B11 | 5.15 ± 0.69 | 15.333** | .711** | .693 | .964 | .522 | .722 |
| B12 | 5.17 ± 0.71 | 14.488** | .714** | .695 | .964 | .515 | .718 |
| B13 | 5.08 ± 0.81 | 13.384** | .676** | .652 | .964 | .452 | .672 |
| B14 | 5.19 ± 0.70 | 16.968** | .694** | .675 | .964 | .494 | .703 |
| B15 | 4.89 ± 0.91 | 12.221** | .639** | .609 | .965 | .380 | .616 |
| B16 | 4.95 ± 0.84 | 14.853** | .697** | .673 | .964 | .468 | .684 |
| B17 | 4.70 ± 1.00 | 14.193** | .691** | .662 | .965 | .451 | .672 |
| B18 | 4.92 ± 0.80 | 14.073** | .773** | .755 | .964 | .603 | .777 |
| B19 | 4.99 ± 0.79 | 15.262** | .728** | .707 | .964 | .537 | .733 |
| B20 | 4.70 ± 1.08 | 14.506** | .646** | .611 | .965 | .386 | .621 |
| B21 | 4.46 ± 1.19 | 11.441** | .566** | .521 | .966 | .282 | .531 |
| B22 | 4.94 ± 0.78 | 16.704** | .745** | .726 | .964 | .562 | .749 |
| B23 | 5.05 ± 0.72 | 16.252** | .743** | .725 | .964 | .574 | .757 |
| B24 | 5.03 ± 0.73 | 16.514** | .781** | .765 | .964 | .628 | .792 |
| B25 | 5.07 ± 0.79 | 17.894** | .746** | .727 | .964 | .580 | .761 |
| B26 | 5.01± 0.79 | 18.578** | .755** | .736 | .964 | .581 | .762 |
| B27 | 5.07 ± 0.72 | 19.765** | .829** | .817 | .964 | .715 | .845 |
| B28 | 5.11 ± 0.71 | 17.638** | .781** | .766 | .964 | .644 | .802 |
| B29 | 5.05 ± 0.71 | 18.881** | .792** | .777 | .964 | .659 | .812 |
| B30 | 5.04 ± 0.73 | 16.005** | .750** | .732 | .964 | .582 | .763 |
| B31 | 5.02 ± 0.72 | 14.959** | .727** | .708 | .964 | .547 | .740 |
| B32 | 5.00 ± 0.73 | 15.684** | .760** | .743 | .964 | .594 | .771 |
| B33 | 5.04 ± 0.76 | 14.285** | .710** | .690 | .964 | .518 | .719 |
| B34 | 5.09 ± 0.75 | 13.815** | .709** | .689 | .964 | .527 | .726 |
| B35 | 5.06 ± 0.74 | 14.658** | .745** | .727 | .964 | .560 | .749 |

C^2^: Communalities coefficient
