# Supplementary material for: Psychometric properties of the Chinese version of the spiritual care-giving scale (C-SCGS) in nursing practice
Source: BMC Med Res Methodol. 2019 Jan 23;19:21. doi: 10.1186/s12874-019-0662-7 (PMC6343288; doi:10.1186/s12874-019-0662-7)
Supplement: Supplementary file 2 — Table S2. The Structure Matrix of the 35-items C-SCGS. (DOCX 24 kb) [file 12874_2019_662_MOESM2_ESM.docx]

**Table S2** The Structure Matrix of the 35-items C-SCGS

| Items | Factor | | | | |
| --- | --- | --- | --- | --- | --- |
|  | 1 | 2 | 3 | 4 | 5 |
| B28 Spiritual care should be positively reinforced in nursing practice.  心灵关怀应在护理实践中得到积极加强 | **.809** | .409 | .475 | .635 | .302 |
| B23 Nurses provide spiritual care by respecting the dignity of patients.  护士通过尊重患者的尊严为其提供心灵关怀 | **.805** | .502 | .568 | .610 | .094 |
| B24 Spiritual care should take into account of what patients think about spirituality.  心灵关怀应考虑到患者的心灵理念 | **.785** | .475 | .619 | .594 | .159 |
| B25 Nurses who are spiritual aware are more likely to provide spiritual care.  具有心灵意识的护士更有可能提供心灵照护 | **.779** | .466 | .526 | .549 | .232 |
| B27 Spiritual care should be instilled throughout a nursing education programme.  心灵关怀的理念应融入护理教育课程 | **.775** | .402 | .506 | .646 | .311 |
| B29 The ability to provide spiritual care develops through experience.  提供心灵关怀的能力通过经历/体验得以发展 | **.751** | .487 | .434 | .527 | .267 |
| B31 Spirituality is influenced by individual’s life experiences.  灵性（心灵）受个人生活经历的影响 | **.734** | .495 | .542 | .552 | .434 |
| B30 Spiritual care is important because it gives patient hope.  心灵关怀因给予患者希望而有价值 | **.728** | .508 | .417 | .571 | .282 |
| B32 Spirituality helps when facing life’s difficulties and problems.  灵性（心灵）助力/帮助面对生活的困难和问题 | **.722** | .469 | .464 | .511 | .402 |
| B26 Spiritual care requires awareness of one's spirituality.  心灵关怀需意识到自身的心灵世界 | **.705** | .527 | .529 | .487 | .159 |
| B35 A team approach is important for spiritual care.  团队的方式对心灵关怀很重要 | **.685** | .386 | .517 | .642 | .529 |
| B34 A trusting nurse-patient relationship is needed to provide spiritual care.  提供心灵关怀需基于信任的护患关系 | **.673** | .327 | .490 | .556 | .561 |
| B22 I am comfortable providing spiritual care to patients.  我舒心地为患者提供心灵关怀 | **.652** | .569 | .381 | .551 | .063 |
| B16 Sensitivity and intuition help the nurse to provide spiritual care.  敏感性和直觉助力护士提供心灵关怀 | **.630** | .608 | .464 | .613 | .265 |
| B21 Spiritual care includes support to help patients observe their religious beliefs.  心灵关怀即支持或帮助患者保持其宗教信仰 | .386 | **.714** | .164 | .221 | .051 |
| B20 Spiritual care enables the patient to find meaning and purpose in their illness.  心灵关怀使患者找到其患病的意义和目的 | .387 | **.685** | .213 | .366 | .140 |
| B18 Nurses provide spiritual care by respecting the religious and cultural beliefs of patients.  护士通过尊重患者的宗教和文化信仰为其提供心灵关怀 | .630 | **.662** | .469 | .551 | .086 |
| B15 Spiritual care is respecting a patient’s religious or personal beliefs.  心灵关怀即尊重患者的宗教或个人信仰 | .409 | **.659** | .374 | .410 | .017 |
| B17 Being with a patient is a form of spiritual care.  心灵关怀的形式之一是与患者在一起 | .381 | **.650** | .276 | .390 | .292 |
| B19 Nurses provide spiritual care by Giving patients time to discuss and explore their fears, anxieties and troubles  护士给予患者足够的时间谈论和探究其恐惧、焦虑和烦恼为其提供心灵关怀 | .621 | **.648** | .455 | .520 | .239 |
| B6 Spirituality is about finding meaning in the good and bad events of life.  灵性是指寻找生活中好坏事件的意义 | .374 | **.573** | .381 | .466 | .206 |
| B3 Spirituality is part of a unifying force which enables individuals to be at peace.  灵性是能使人平和/安宁、和睦的达成一致的力量部分 | .569 | .368 | **.768** | .607 | .191 |
| B4 Spirituality is an expression of one’s inner feelings that affect behaviour.  灵性是一种影响人的行为的内在情感表达 | .544 | .365 | **.753** | .564 | .199 |
| B2 Spirituality is an important aspect of human beings.  灵性（心灵）是人类的一个重要方面 | .469 | .224 | **.716** | .473 | .200 |
| B5 Spirituality is part of our inner being.  灵性是我们内心的一部分 | .456 | .393 | **.676** | .535 | .389 |
| B1 Everyone has spirituality.  每个人都有灵性（心灵） | .310 | .213 | **.629** | .395 | .223 |
| B8 Spirituality drives individuals to search for answers about meaning and purpose in life.  灵性驱使个人寻找生活的意义及其目的的答案 | .623 | .455 | .555 | **.760** | .198 |
| B12 Spiritual care is more than religious care.  心灵关怀不只是宗教的关怀 | .571 | .392 | .577 | **.735** | .276 |
| B11 Spiritual care is an integral component of holistic nursing care.  心灵关怀是整体护理的重要组成 | .591 | .411 | .519 | **.727** | .251 |
| B10 Spiritual needs are met by connecting oneself with other people, higher power or nature.  灵性的需求通过自己与他人、更大能量或自然界的联系得到满足 | .561 | .517 | .621 | **.709** | .257 |
| B7 Spiritual well-being is important for one’s emotional well-being.  心灵的幸福对个体的情感健康很重要 | .549 | .272 | .642 | **.664** | .211 |
| B14 Spiritual care is a process and not a one- time event or activity.  心灵关怀是个过程，而不是一次性事件或活动 | .586 | .348 | .551 | **.658** | .159 |
| B13 Nursing care, when performed well, is itself, spiritual care.  良好的护理本身就是心灵关怀 | .600 | .524 | .443 | **.652** | .095 |
| B9 Spiritual needs are met by connecting oneself with other people, higher power or nature.  没有灵性,就不是个完整的人 | .380 | .488 | .398 | **.575** | .252 |
| B33 Spiritual care requires the nurse to be empathetic towards the patient.  心灵关怀要求护士对患者富有同情心 | .632 | .433 | .508 | .515 | **.702** |
| Extraction Method: Principal Axis Factoring.  Rotation Method: Promax with Kaiser Normalization. | | | | | |
