# Supplementary figures and images for: Psychometric properties of the Chinese version of the spiritual care-giving scale (C-SCGS) in nursing practice
Source: BMC Med Res Methodol. 2019 Jan 23;19:21. doi: 10.1186/s12874-019-0662-7 (PMC6343288; doi:10.1186/s12874-019-0662-7)

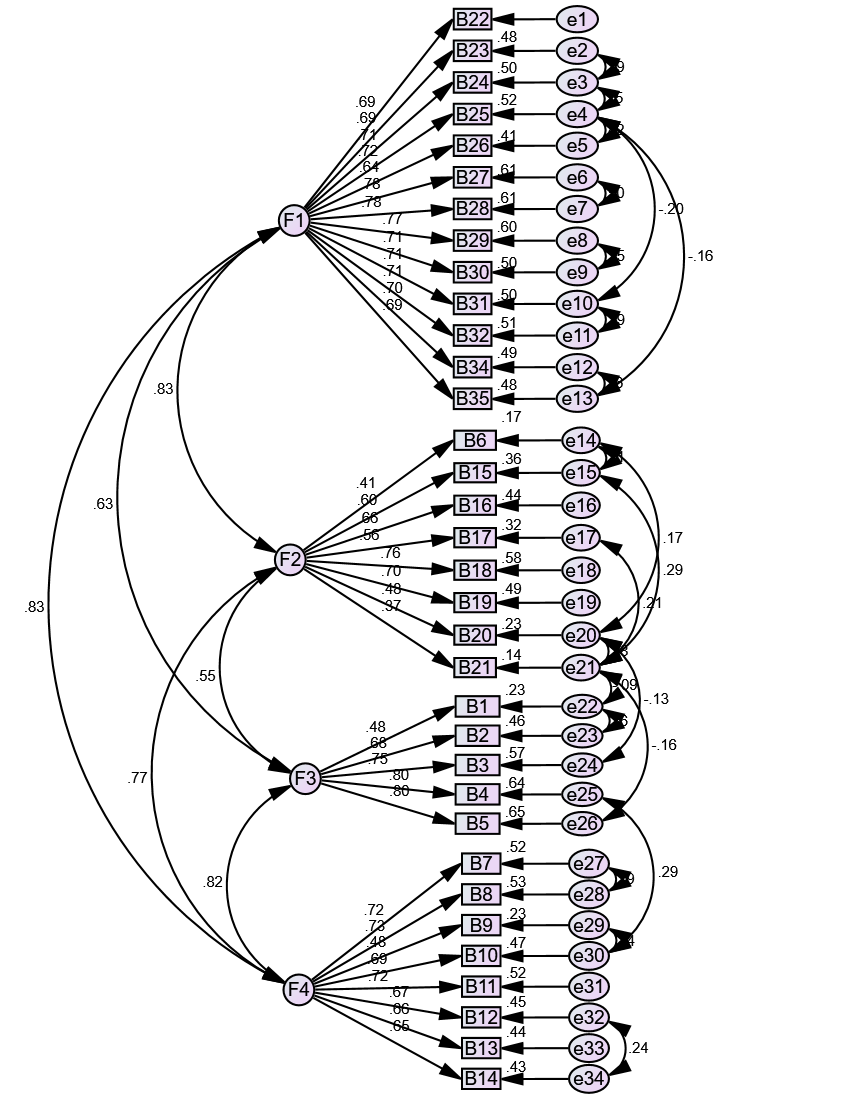


**Figure S1** Results of the confirmatory factor analysis

Supplement: Supplementary file 3 — Figure S1. Results of the confirmatory factor analysis. (DOCX 253 kb) [file 12874_2019_662_MOESM3_ESM.docx]
