# Supplementary material for: Psychometric properties of the Chinese version of the spiritual care-giving scale (C-SCGS) in nursing practice
Source: BMC Med Res Methodol. 2019 Jan 23;19:21. doi: 10.1186/s12874-019-0662-7 (PMC6343288; doi:10.1186/s12874-019-0662-7)
Supplement: Supplementary file 4 — The Chinese version of the Spiritual Care-Giving Scale. (DOCX 20 kb) [file 12874_2019_662_MOESM4_ESM.docx]

Additional file 5: 中文版灵性照护量表the Chinese version of the Spiritual Care-Giving Scale

对于每一项，请选择最能反映你同意或不同意程度的答案

|  | **条目Items** | **非常不同意**  Strongly Disagree | **不同意**  Disagree | **稍不同意**  Mildly Disagree | **稍同意**  Mildly Agree | **同意**  Agree | **非常同意**  Strongly Agree |
| --- | --- | --- | --- | --- | --- | --- | --- |
| 1 | 每个人都有灵性（心灵）  Everyone has spirituality. |  |  |  |  |  |  |
| 2 | 灵性是人类的一个重要方面  Spirituality is an important aspect of human beings. |  |  |  |  |  |  |
| 3 | 灵性是能使人平和/安宁、和睦的达成一致的力量部分  Spirituality is part of a unifying force which enables individuals to be at peace |  |  |  |  |  |  |
| 4 | 灵性是一种影响人的行为的内在情感表达  Spirituality is an expression of one’s inner feelings that affect behaviour. |  |  |  |  |  |  |
| 5 | 灵性是我们内心的一部分  Spirituality is part of our inner being. |  |  |  |  |  |  |
| 6 | 灵性是指寻找生活中好坏事件的意义  Spirituality is about finding meaning in the good and bad events of life |  |  |  |  |  |  |
| 7 | 心灵的幸福对个体的情感健康很重要  Spiritual well-being is important for one’s emotional well-being |  |  |  |  |  |  |
| 8 | 灵性驱使个人寻找生活的意义及其目的的答案  Spirituality drives individuals to search for answers about meaning and purpose in life. |  |  |  |  |  |  |
| 9 | 没有灵性,就不是个完整的人  Without spirituality, a person is not considered whole. |  |  |  |  |  |  |
| 10 | 灵性的需求通过自己与他人、更大能量或自然界的联系得到满足  Spiritual needs are met by connecting oneself with other people, higher power or nature. |  |  |  |  |  |  |
| 11 | 心灵关怀是整体护理的重要组成  Spiritual care is an integral component of holistic nursing care |  |  |  |  |  |  |
| 12 | 心灵关怀不只是宗教的关怀  Spiritual care is more than religious care. |  |  |  |  |  |  |
| 13 | 良好的护理本身就是心灵关怀  Nursing care, when performed well, is itself, spiritual care. |  |  |  |  |  |  |
| 14 | 心灵关怀是个过程，而不是一次性事件或活动  Spiritual care is a process and not a one- time event or activity. |  |  |  |  |  |  |
| 15 | 心灵关怀即尊重患者的宗教或个人信仰  Spiritual care is respecting a patient’s religious or personal beliefs |  |  |  |  |  |  |
| 16 | 敏感性和直觉助力护士提供心灵关怀  Sensitivity and intuition help the nurse to provide spiritual care. |  |  |  |  |  |  |
| 17 | 心灵关怀的形式之一是与患者在一起  Being with a patient is a form of spiritual care. |  |  |  |  |  |  |
| 18 | 护士通过尊重患者的宗教和文化信仰为其提供心灵关怀  Nurses provide spiritual care by respecting the religious and cultural beliefs of patients. |  |  |  |  |  |  |
| 19 | 护士给予患者足够的时间谈论和探究其恐惧、焦虑和烦恼为其提供心灵关怀  Nurses provide spiritual care by Giving patients time to discuss and explore their fears, anxieties and troubles |  |  |  |  |  |  |
| 20 | 心灵关怀使患者找到其患病的意义和目的  Spiritual care enables the patient to find meaning and purpose in their illness |  |  |  |  |  |  |
| 21 | 心灵关怀即支持或帮助患者保持其宗教信仰  Spiritual care includes support to help patients observe their religious beliefs |  |  |  |  |  |  |
| 22 | 我舒心地为患者提供心灵关怀  I am comfortable providing spiritual care to patients. |  |  |  |  |  |  |
| 23 | 护士通过尊重患者的尊严为其提供心灵关怀  Nurses provide spiritual care by respecting the dignity of patients. |  |  |  |  |  |  |
| 24 | 心灵关怀应考虑到患者的心灵理念  Spiritual care should take into account of what patients think about spirituality |  |  |  |  |  |  |
| 25 | 具有心灵意识的护士更有可能提供心灵照护  Nurses who are spiritual aware are more likely to provide spiritual care. |  |  |  |  |  |  |
| 26 | 心灵关怀需意识到自身的心灵世界  Spiritual care requires awareness of one's spirituality |  |  |  |  |  |  |
| 27 | B27 心灵关怀的理念应融入护理教育课程  Spiritual care should be instilled throughout a nursing education programme |  |  |  |  |  |  |
| 28 | 心灵关怀应在护理实践中得到该积极加强  Spiritual care should be positively reinforced in nursing practice. |  |  |  |  |  |  |
| 29 | 提供心灵关怀的能力通过经历/体验得以发展  The ability to provide spiritual care develops through experience. |  |  |  |  |  |  |
| 30 | 心灵关怀因给予患者希望而有价值  Spiritual care is important because it gives patient hope |  |  |  |  |  |  |
| 31 | 灵性（心灵）受个人生活经历的影响  Spirituality is influenced by individual’s life experiences. |  |  |  |  |  |  |
| 32 | 灵性（心灵）助力/帮助面对生活的困难和问题  Spirituality helps when facing life’s difficulties and problems. |  |  |  |  |  |  |
| 33 | 提供心灵关怀需基于信任的护患关系  A trusting nurse-patient relationship is needed to provide spiritual care |  |  |  |  |  |  |
| 34 | 团队的方式对心灵关怀很重要  A team approach is important for spiritual care |  |  |  |  |  |  |
